# Wearable plasmonic paper–based microfluidics for continuous sweat analysis

**DOI:** 10.1126/sciadv.abn1736

**Published:** 2022-03-23

**Authors:** Umesha Mogera, Heng Guo, Myeong Namkoong, Md Saifur Rahman, Tan Nguyen, Limei Tian

**Affiliations:** Department of Biomedical Engineering, Center for Remote Health Technologies and Systems, Texas A&M University, College Station, TX 77843, USA.

## Abstract

Wearable sweat sensors have the potential to provide clinically meaningful information associated with the health and disease states of individuals. Current sensors mainly rely on enzymes and antibodies as biorecognition elements to achieve specific quantification of metabolite and stress biomarkers in sweat. However, enzymes and antibodies are prone to degrade over time, compromising the sensor performance. Here, we introduce a wearable plasmonic paper–based microfluidic system for continuous and simultaneous quantitative analysis of sweat loss, sweat rate, and metabolites in sweat. Plasmonic sensors based on label-free surface-enhanced Raman spectroscopy (SERS) can provide chemical “fingerprint” information for analyte identification. We demonstrate the sensitive detection and quantification of uric acid in sweat at physiological and pathological concentrations. The well-defined flow characteristics of paper microfluidic devices enable accurate quantification of sweat loss and sweat rate. The wearable plasmonic device is soft, flexible, and stretchable, which can robustly interface with the skin without inducing chemical or physical irritation.

## Teaser

A wearable plasmonic microfluidic device enables continuous quantification of sweat loss, sweat rate, and metabolites.

## INTRODUCTION

Soft, ultrathin skin-interfaced physiological sensors for continuous measurement of physical and chemical biomarkers have broad applications, including disease diagnosis, health monitoring, and personalized medicine (*1*–*6*). Recently developed wearable sweat sensors can analyze various chemicals in sweat, including electrolytes, metabolites, heavy metals, drugs, and hormones, which can reflect physiological and pathological conditions in the human body (*7*–*11*). For example, sweat chloride concentration is a standard diagnostic screening parameter for cystic fibrosis, and the quantification of sweat glucose has been extensively explored for diabetes management (*12*, *13*). Similarly, uric acid (UA) is a risk biomarker for various diseases, including cardiovascular diseases, kidney diseases, and type 2 diabetes (*14*–*16*). It has been shown that sweat UA concentration is highly correlated with serum concentrations in healthy subjects and patients with gout (*17*). To accurately quantify these biomarkers, wearable sweat sensors require high sensitivity, specificity, and mechanical and environmental stability. Existing sensing modalities mainly rely on electrochemical and colorimetric approaches. These sensors mainly rely on enzymes and antibodies as biorecognition elements to achieve specific quantification of metabolite and stress biomarkers in sweat (*1*, *7*, *18*–*20*). For example, enzymes were used for the specific detection of glucose, lactate, UA, urea, and ascorbic acid (*10*, *11*, *18*, *21*). Antibodies were used for the specific detection of cortisol, a stress biomarker (*19*, *22*). However, enzymes and antibodies are prone to degrade over time and lose their functionality after exposure to harsh environments and contamination (*23*–*26*). Therefore, continuous measurements of chemical analytes with high sensitivity, selectivity, and environmental stability remain challenging.

Surface-enhanced Raman spectroscopy (SERS) is a highly sensitive analytical method for label-free detection and quantification of a wide range of analytes, including metabolites, macromolecules, and microorganisms (*27*, *28*). Raman bands of analytes originate from vibrational and rotational modes specific to the molecular structures, which provide chemical “fingerprint” information for analyte identification (*29*). However, Raman scattering is very weak as only one in 10^6^ to 10^10^ photons are scattered inelastically. Plasmonic nanostructures can greatly enhance the Raman scattering of analytes near the nanostructure surface by factors of 10^8^ or higher, which enables single-molecule detection (*30*–*33*). Up to date, substantial progress has been made in the fabrication of various SERS substrates to provide high sensitivity, uniformity, and stability for reliable quantification of analytes at a very low concentration (*27*, *34*). To further improve sample collection efficiency, flexible SERS substrates, such as plasmonic paper and foams, have been developed (*24*, *26*, *35*–*43*). Recent works show that SERS offers highly sensitive and specific trace detection in sweat using silver nanowires or silver nanocubes as SERS substrates (*35*, *36*). However, silver is not chemically and environmentally stable, resulting in decreased SERS performance over time, thereby not ideal for wearable applications (*44*, *45*). In addition, a microfluidic sensing platform integrated with plasmonic sensors has not been reported for continuous and simultaneous sweat rate and composition analysis. Simultaneous quantification of sweat rate and biomarker concentration is essential because these two parameters are intrinsically linked in sweat secretion and reabsorption processes. For instance, the concentration of sodium, chloride, urea, and creatinine changes with changing sweat rate (*46*, *47*).

In this work, we introduce a wearable plasmonic paper–based microfluidic (paperfluidic) system that can directly and reliably capture sweat and continuously and simultaneously quantify sweat loss, sweat rate, and the concentration of analytes in sweat in real time. The paper microfluidics enables accurate quantification of sweat loss and sweat rate. The integrated plasmonic nanosensors can detect and quantify UA at physiologically and pathologically relevant concentrations using SERS. The ratiometric SERS approach can reliably quantify UA with variation in the laser power and focus, validated with benchtop and portable Raman spectrometers. We demonstrate two operation modes of using plasmonic paperfluidic devices to quantify the analytes of varying concentrations, including in situ continuous scans and batch analysis by scanning the samples at the endpoint. The device is soft, thin, flexible, and stretchable, which can interface to the skin without inducing chemical or physical irritation.

## RESULTS

### Design of wearable plasmonic paperfluidic device for continuous sweat analysis

**Fig. 1.**
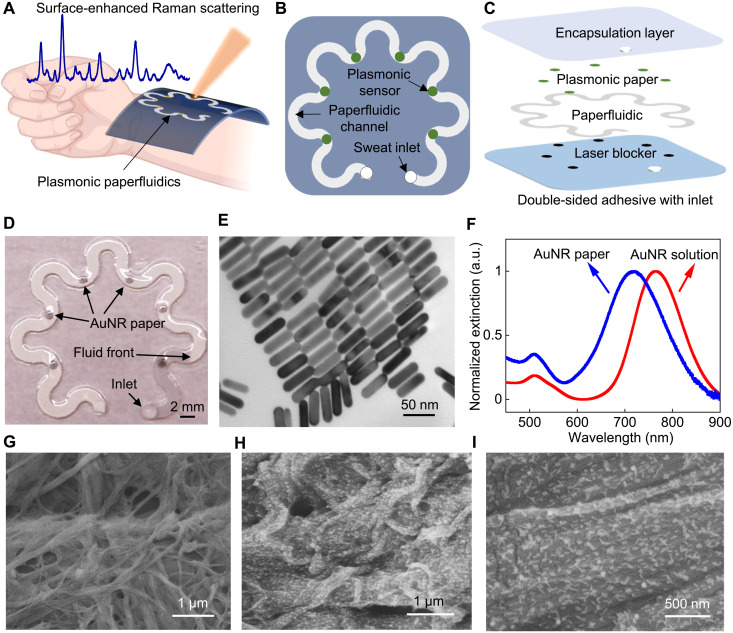
Wearable plasmonic paper microfluidic device. (**A**) Conceptual illustration of a wearable plasmonic paperfluidic device for sweat collection, storage, and in situ analysis using SERS. (**B**) Top view and (**C**) stacked view schematics of the paperfluidic device highlighting key functional layers. (**D**) Photograph of an assembled paperfluidic device with six plasmonic sensors. (**E**) TEM image of gold nanorods (AuNRs) with a uniform size distribution. (**F**) Extinction spectra of AuNR solution and AuNR paper. a.u., arbitrary units. SEM images of pristine chromatography paper (**G**) and AuNR paper (**H** and **I**).

Figure 1A shows a concept illustration of a soft, ultrathin plasmonic paperfluidic device laminated on the wrist for sweat collection, transport, storage, and real-time label-free biochemical analysis with a portable Raman spectrometer. The paper microfluidic devices have several advantages, including (i) cost-effective and easy disposal, (ii) simple capture and transportation of biofluids through capillary action without the need of an additional pump or force, (iii) absorbency allowing the storage of sensors and samples, (iv) air permeability avoiding air bubble problems, and (v) high surface area allowing a high-density immobilization of nanoparticles. The wearable plasmonic sweat sensors comprise several functional layers, including a double-sided adhesive, laser blocker, paper microfluidic layer, plasmonic sensors, and top encapsulation layer (Fig. 1, B and C). A cellulose chromatography paper with a serpentine design serves as an effective microfluidic channel that transports the excreted sweat through the porous medium by wicking without the need for external force or inlet pressure. The serpentine design provides paper microfluidic devices flexibility and stretchability and allows the device to accommodate the skin deformation without causing interfacial stress and device degradation. A stretchable double-sided adhesive forms a mechanically robust interface between the paper microfluidic layer and the skin. A large inlet opening is beneficial to maximize sweat collection by increasing access to sweat glands. However, this creates a dead volume, inducing a deviation in real-time sweat rate quantification. Therefore, in our design, the diameter of the inlet opening follows the width of the microfluidic paper. In addition, the well-encapsulated microfluidic paper with a small inlet opening isolates and directs sweat along a controlled channel to interact with sensors and minimizes external contamination. An outlet with the same size as the inlet is located at the end of the paperfluidic channel to avoid backpressure. Plasmonic sensors immobilized at different locations along the paper microfluidic channel quantify the concentration of analytes in sweat produced at different time points using Raman spectroscopy. Black carbon double-sided adhesive blocks laser and avoids skin damage during in situ Raman spectroscopy measurements. The top polydimethylsiloxane (PDMS) encapsulation layer is optically transparent and exhibits well-defined Raman bands, which serves as a reference for quantifying the analytes of interest in sweat. In addition, it minimizes the evaporation of sweat and prevents contamination from the environment.

Figure 1D shows a photograph of an assembled device with the inlet on the encapsulation side for demonstration. A droplet of 10 μl of water was added to the inlet, and the fluid passed the first plasmonic sensor within a minute. The color contrast between the wetted and dry regions is clearly disguisable, allowing the quantification of sweat volume and sweat rate with the position of the fluid front. Plasmonic sensors comprise the chromatography paper uniformed adsorbed with gold nanorods (AuNRs), described as AuNR paper in the following discussion. Silver and gold nanostructures are commonly used in SERS due to the enhanced electromagnetic field near the nanostructure surface (*27*, *34*). Silver nanostructures typically provide higher SERS enhancement and are more cost-effective than gold nanostructures. However, silver is not chemically and environmentally stable, resulting in decreased SERS performance over time (*44*, *45*). In our design, we choose AuNR paper as a SERS substrate for stable SERS enhancement. Gold nanostructures are chemically and environmentally stable. AuNRs offer higher SERS enhancement compared to other gold nanostructures such as gold nanospheres (*27*).

We synthesized AuNRs using a seed-mediated method, as described in previous work (*48*, *49*). Tuning the localized surface plasmon resonance (LSPR) wavelength of AuNRs to overlap with the laser excitation wavelength generates much higher SERS intensity than those without overlapping bands (*50*). In our design, we chose the AuNRs with the longitudinal LSPR wavelength of 765 nm to overlap with the laser excitation wavelength of 780 or 785 nm in benchtop and portable spectrometers (fig. S7B). The immobilization of AuNRs onto the paper is facilitated by the combination of weak interactions, including electrostatic interaction and van der Waals forces (*43*, *51*). The synthesized AuNRs exhibit a uniform dimension distribution of 56.9 ± 3.6 nm in length and 14.5 ± 1.5 nm in diameter, respectively (Fig. 1E and fig. S1). The extinction of the AuNR solution shows two plasmonic bands with peak positions at 511.5 and 765.0 nm, corresponding to the transverse and longitudinal LSPR of AuNRs. The LSPR spectrum of AuNR paper shows a 48.3-nm blue shift in the longitudinal band following the decrease in the refractive index of surrounding media from water to air and cellulose. The shape of the extinction spectrum of AuNR paper remains similar to that of the solution, which suggests the uniform distribution of AuNRs on the paper substrate. The uniformity is further confirmed by scanning electron microscopy (SEM). The SEM image of the pristine paper reveals the heterogeneous morphology of cellulose fibers with diameters from 100 nm to 2 μm (Fig. 1G). The SEM images of AuNR paper show uniform distribution of AuNRs on the heterogeneous paper surface (Fig. 1, H and I), which is critical for achieving uniform Raman signals from SERS substrates.

### Flow characteristics of paper microfluidic devices for sweat loss/rate quantification

**Fig. 2.**
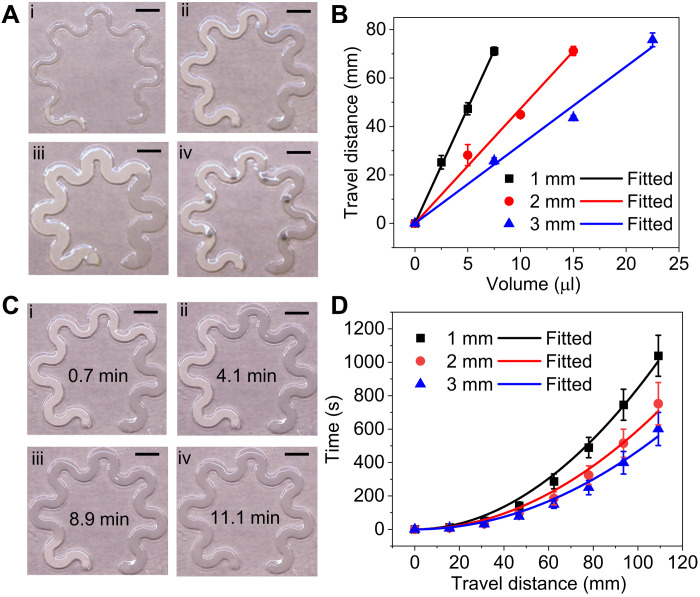
Flow characteristics of paper microfluidic devices. (**A**) Photographs of serpentine paperfluidic devices with different paper widths of (i) 1 mm, (ii) 2 mm, (iii) 3 mm, and (iv) 2 mm with integrated plasmonic sensors, showing varying travel distances of 10 μl of water. (**B**) Travel distance as a function of the input liquid volume. (**C**) Photographs of a 2-mm-wide paperfluidic device showing the increasing liquid travel distance over time from 0.7 to 11.1 min. (**D**) Relationship between liquid travel time and travel distance in the microfluidic paper with varying channel widths. The solid lines are fitted curves of the experimental data (dots). Scale bars, 5 mm.

For sweat volume and sweat rate quantification, we characterize the flow characteristics of the microfluidic serpentine paper sandwiched between top encapsulation PDMS and bottom adhesive layers. We hypothesize that the fluid uptake volume of the microfluidic paper is linearly proportional to the paper width and liquid travel distance for a given paper thickness. We measured the travel distance of water with varying known volumes along the serpentine paper with a central length of 109 mm, a thickness of 180 μm, and varying widths of 1, 2, and 3 mm. For 10 μl of water introduced to the inlet, the travel distance is 95, 47, and 32 mm for the paper width of 1, 2, and 3 mm, respectively (Fig. 2A, i to iii). The integration of AuNR paper with a diameter of 1 mm has a negligible effect on the liquid travel distance (Fig. 2Aiv). Figure 2B shows the increasing linear trend of travel distance with the increase in the liquid volume for all the samples. The calculated volume and travel distance relationship is 9.52 ± 0.09 mm/μl, 4.74 ± 0.16 mm/μl, and 3.24 ± 0.12 mm/μl for the paper with channel widths of 1, 2, and 3 mm, respectively. The total liquid uptake volume of the 1-, 2-, and 3-mm-wide paper is calculated to be 11.5 ± 0.1 μl, 23.0 ± 0.8 μl, and 33.7 ± 1.2 μl, respectively. One can use these calibrated values to quantify the sweat volume by visualizing the position of the fluid front.

To characterize the liquid wicking kinetics of microfluidic paper, video recordings of continuous flow quantify the travel distance and corresponding time (fig. S2, A to C). Figure 2C demonstrates the increased liquid travel distance in the 2-mm-wide serpentine paper following the increasing flow time from 0.7 to 11.1 min. We measured the travel time across varying distances in paperfluidic devices with channel widths of 1 to 3 mm (Fig. 2D and fig. S2, D to F). The travel time across the same length decreases with the increase in the paper channel width. In comparison, the travel time across the entire length of 109 mm is 16.9 min for 1-mm-wide paper, 11.7 min for 2-mm-wide paper, and 8.9 min for 3-mm-wide paper. We also measured the flow rate under the relative humidity of 45 and 90% and found that humidity had a negligible effect (fig. S3). The fluid flow in the microfluidic paper can be modeled by the Lucas-Washburn equation, $l=\sqrt{\frac{\gamma d\text{cosθ}t}{4\mu }}$, in which *l* represents the travel distance, γ is the surface tension of the liquid, *d* is the average pore radius, μ is the viscosity of the liquid, θ is the contact angle between the fluid and the boundary wall, and *t* represents time (*52*). The fitted $\sqrt{\frac{\gamma d\text{cosθ}}{4\mu }}$ is 3.44 mm/s^1/2^ for 1-mm-wide paper, 4.10 mm/s^1/2^ for 2-mm-wide paper, and 4.62 mm/s^1/2^ for 3-mm-wide paper. Although the wicking rate of the microfluidic paper slows down with increasing travel distance, with excess fluid supply in the inlet, the active wicking rate is much higher than the typical sweat rate of 12 to 120 μl cm^2^ hour^−1^ (*53*). For the 2-mm-diameter sweat collection area, the sweat rate is as high as 0.06 μl/min. The wicking rate of the 2-mm-wide microfluidic paper close to the outlet is 0.98 μl/min, 15.5 times higher than the potential maximum sweat rate. Therefore, the wicking rate can be used to quantify real-time sweat rate. On the basis of these results, the specific dimension of the microfluidic paper can be chosen to quantify the sweat volume and sweat rate depending on the mounting location of the device on the body.

### Plasmonic paperfluidic device design and optimization

**Fig. 3.**
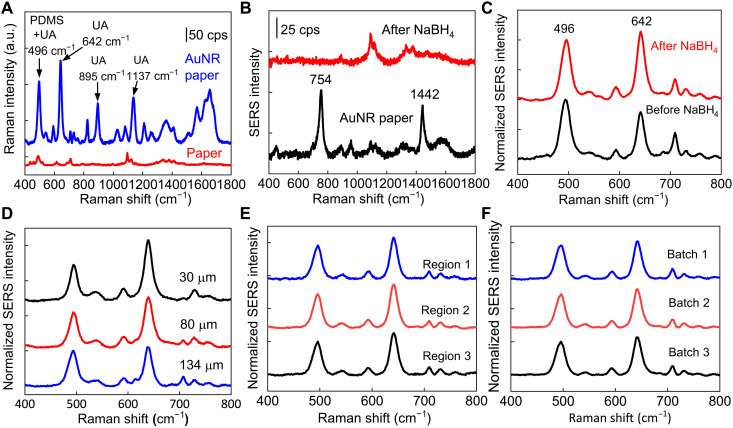
Plasmonic sensor optimization. (**A**) Raman spectra of 100 μM UA in 1× PBS collected on pristine paper and AuNR paper. cps, counts per second. (**B**) Raman spectra of AuNR paper before and after removing surfactants from AuNR with NaBH_4_, and (**C**) corresponding SERS spectra of UA collected from AuNR paper. (**D**) SERS spectra of 100 μM UA with a PDMS encapsulation layer of varying thicknesses. SERS spectra of 100 μM UA collected from (**E**) different regions of AuNR paper in the same batch and (**F**) different batches of AuNR paper.

Next, we designed and characterized AuNR paper sandwiched between a microfluidic paper and top encapsulation PDMS for highly sensitive detection of UA using SERS spectroscopy. Figure 3A shows Raman spectra of 100 μM UA in 1× phosphate-buffered saline (PBS) collected on AuNR paper and pristine paper without AuNR. The Raman bands of UA measured from AuNR paper are clearly distinguishable due to the enhancement effect of AuNR, while the signals are not detectable from the pristine paper. The prominent peaks observed at 642, 895, and 1137 cm^−1^ correspond to the skeletal ring deformation, N-H bending, and C-N stretching of UA (*54*, *55*). These Raman bands are consistent with these measured from UA powder (fig. S4A). The peak at 496 cm^−1^ is contributed from the C-N bending and in-plane ring deformation of UA and the Si-O-Si stretch of PDMS (fig. S4B) (*56*). To maximize the SERS enhancement of UA and minimize the interference from AuNR paper, we used sodium borohydride (NaBH_4_) to remove cetyltrimethylammonium bromide (CTAB) on the AuNR surface. CTAB plays an important role in the growth and stabilization of AuNRs in an aqueous solution (*48*, *49*). After AuNR immobilization on paper and extensive water rinsing, strong Raman bands of CTAB with peaks at 754 and 1442 cm^−1^ are still present because of strong interaction between Br^−^ and Au. The residual CTAB can be rapidly removed by 10 mM NaBH_4_ within 10 min (Fig. 3B). The removal of CTAB stabilizer has a negligible effect on the distribution of AuNR on paper, confirmed with extinction spectra of AuNR paper before and after NaBH_4_ treatment (fig. S5).

To quantify the UA concentration, we use the SERS intensity ratio of UA and PDMS to minimize the variations in the absolute intensity induced by the variations of laser focus. The SERS spectra of UA collected on AuNR paper covered with a quartz slide quantifies that the intensity of 496 cm^−1^ peak is 0.4 times as that of 642 cm^−1^ peak (fig. S6). Therefore, a ratiometric intensity *I*_642_/(*I*_496_ − 0.4 × *I*_642_) is used to quantify the sensitivity of UA detection in biofluids. The removal of CTAB increases the ratiometric SERS intensity of UA by 86% (Fig. 3C). The thickness of the PDMS encapsulation layer also affects the ratiometric SERS intensity of UA, which increases by 1.9 times with the decreased PDMS thickness from 134 to 25 μm (Fig. 3D). We chose PDMS films of 80 μm thick as an encapsulation layer to provide conformal contact with the paper microfluidic channel while minimizing the sweat evaporation. The SERS intensity also increases with increasing the density of AuNRs on the paper substrate by increasing the AuNR adsorption time or the concentration of AuNR solution. For example, the SERS ratiometric intensity increases from 2.0, 2.4, to 4.2 when the extinction intensity of AuNR solutions increases from 2.5, 5, to 10 (fig. S7A). In addition, we quantified the SERS ratiometric intensity to be 4.0 with the AuNR paper prepared using AuNR solution with the longitudinal LSPR wavelength of 650 nm and the extinction intensity of 5 (fig. S7, B and C). The unexpected high SERS intensity from AuNR 650 nm might result from the plasmonic coupling effect between AuNRs, considering that the LSPR wavelength is off-resonant with the laser excitation at 780 or 785 nm (*57*). In the following experiments, we used AuNR paper prepared with AuNR solution with the longitudinal LSPR wavelength of 765 nm and an extinction intensity of 5. We have collected SERS spectra of UA from three different regions of AuNR paper prepared in one batch shown in Fig. 3E and fig. S8A. The dimension of AuNR paper in each batch is 2 cm by 2 cm, prepared by immersing the paper in 0.5 ml of AuNR solution for 15 hours (fig. S9). The SERS spectra shown in Fig. 3F and fig. S8B were collected from three independently prepared batches. The ratiometric SERS intensities of UA collected from different regions of the same sample and from samples of different batches yield coefficients of variations of 4 and 3%, respectively, confirming the uniformity of SERS signals (Fig. 3, E and F, and fig. S8).

### Sensitive and specific UA detection and continuous quantification

**Fig. 4.**
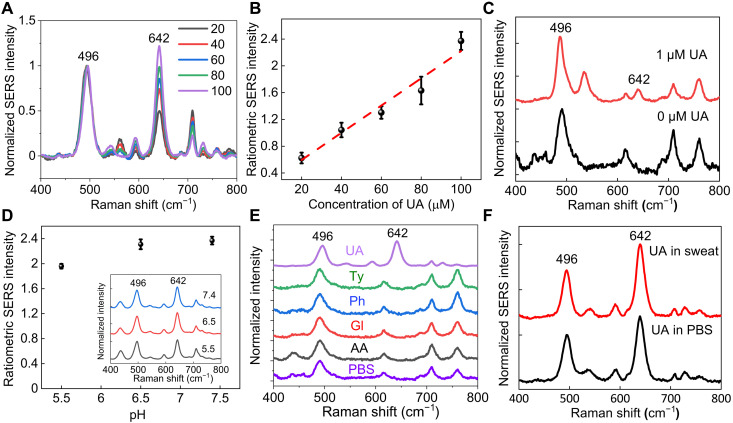
Sensitivity and specificity of UA detection and quantification. (**A**) SERS spectra of UA of varying concentrations averaged from six spectra for each concentration. (**B**) Calibration curve of ratiometric SERS intensity for UA quantification. (**C**) SERS spectra of 0 and 1 μM UA collected from AuNR paper. (**D**) Ratiometric SERS intensity of 100 μM UA at different pH conditions. Inset shows corresponding SERS spectra. (**E**) SERS spectra of 100 μM UA, tyrosine (Ty), phenylalanine (Ph), glucose (Gl), ascorbic acid (AA), and 1× PBS. (**F**) SERS spectra of UA in 1× PBS and artificial sweat.

Figure 4A shows the normalized SERS spectra of UA with varying concentrations from 20 to 100 μM in 1× PBS collected from AuNR paper. The concentration range covers the physiological and pathological concentrations in sweat for healthy people and people with gout and hyperuricemia (*17*). The ratiometric SERS intensity of UA linearly increases with the increase in the concentration (Fig. 4B). The SERS signals were obtained from the concentration of UA as low as 1 μM (Fig. 4C). We evaluated the ratiometric SERS intensity of UA with varying pH over a medically relevant range from pH 5.5 to 7.4 (Fig. 4D). The ratiometric intensity of UA measured at pH 5 and 6.5 compared to that at pH 7.4 decreased by 17 and 3%, respectively. To evaluate the specificity of SERS signals for UA detection, we expose AuNR paper to several potential interfering molecules, including tyrosine, glucose, ascorbic acid, and phenylalanine, at the concentration of 100 μM (Fig. 4E). The tested interfering molecules of 100 μM are within or higher than the physiologically relevant concentrations of 5.6 μM to 2.2 mM glucose, 66 to 300 μM tyrosine, 0.11 to 36 μM ascorbic acid, and 61 to 210 μM phenylalanine (*58*). The SERS spectra of these interfering molecules confirm the absence of Raman bands at 642 cm^−1^; therefore, their presence in biofluids does not affect the accuracy of UA quantification. We further confirmed the specific detection of UA by exposing AuNR paper to 100 μM UA in artificial sweat, which consists of amino acids, minerals, and various metabolites and simulates the composition and properties of the real human eccrine sweat. The ratiometric intensity of UA in PBS and artificial sweat shows negligible difference confirming the specificity (Fig. 4F).

**Fig. 5.**
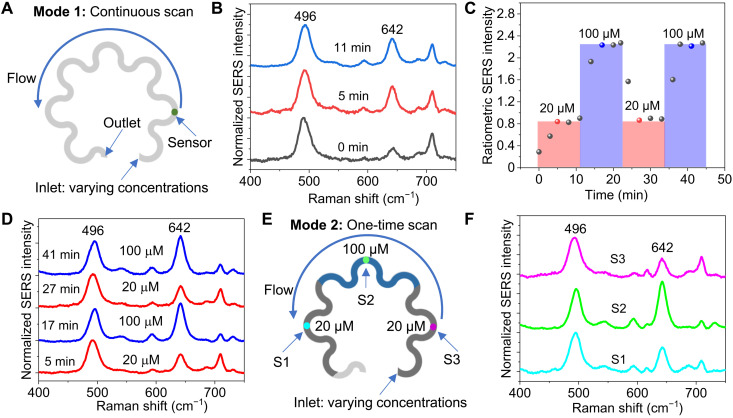
Operation modes of plasmonic paperfluidic devices. (**A**) Illustration of continuously scanning one sensor to quantify the rapidly changing concentration of UA. (**B**) SERS spectra of 20 μM UA collected after the UA solution wetted the sensor. (**C**) Ratiometric SERS intensity of UA with time and with changes in the concentration of UA. (**D**) Representative SERS spectra of UA at the time points indicated as red and blue dots in (C). (**E**) Illustration of scanning multiple sensors at the endpoint, where the device allows for sample storage and batch analysis. (**F**) SERS spectra of UA collected from three sensors, S1, S2, and S3, shown in (E), exposed to varying concentrations of UA.

We demonstrate two operation modes of using plasmonic paperfluidic devices to quantify the analytes of varying concentrations. The first mode involves a continuous scan of one sensor to quantify the rapidly changing concentration of UA (Fig. 5A). In continuous scan mode, the fluid uptake volume can be extended by interfacing the outlet of the paperfluidic device to a cellulose wicking pad. The UA solutions of 30 μl with changing concentrations of 20 and 100 μM were sequentially introduced to the inlet of the paperfluidic device, while the SERS spectra were continuously collected from the sensor. The first spectrum at 0 min was collected right after the 20 μM UA solution wetted the sensor, which showed a weak 642 cm^−1^ peak (Fig. 5B). The ratiometric SERS intensity of UA increased with time and reached a plateau within 5 min (Fig. 5C). After 10 min, the 100 μM UA solution was introduced, and the ratiometric SERS intensity increased and reached another plateau within 5 min (Fig. 5C). Next, another cycle of 20 and 100 μM UA was introduced, and the change in the ratiometric intensity is consistent with the change in the UA concentration (Fig. 5, C and D).

In the second operation mode, the device consists of multiple sensors and allows for sample storage and batch analysis by scanning the samples at the endpoint. To demonstrate this capability, we introduced 10 μl of 20, 100, and 20 μM UA in sequence to the inlet of a plasmonic paperfluidic device (Fig. 5E). Plasmonic sensors S1, S2, and S3 are spatially distributed along the paperfluidic channel to quantify the concentration of sequentially introduced UA solutions. The change in the ratiometric SERS intensity follows the change in the UA concentration, confirming the capability of quantifying the time-varying UA concentration (Fig. 5F).

### Stretchability, flexibility, stability, and application of plasmonic paperfluidic devices

**Fig. 6.**
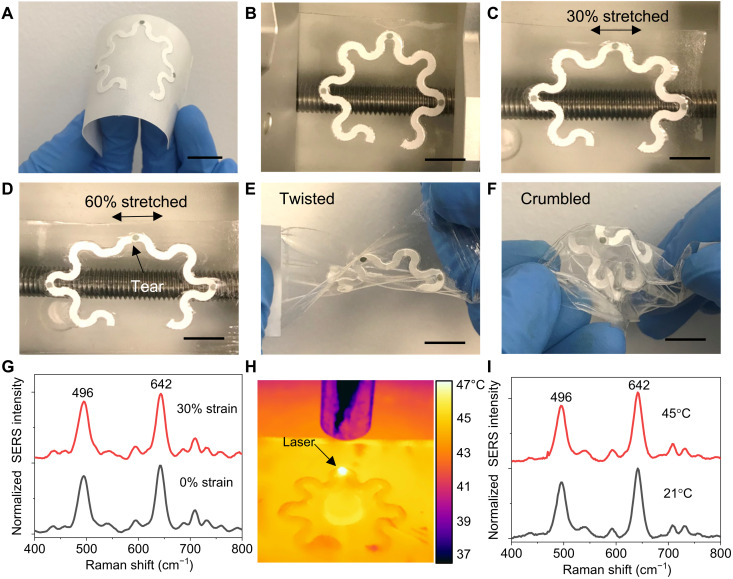
Device flexibility, stretchability, and stability. Photographs of (**A**) a plasmonic paperfluidic device assembled on a medical adhesive with a paper liner, (**B**) a freestanding paperfluidic device mounted on a mechanical stretcher at rest state, (**C**) under 30% stretch, (**D**) under 60% stretch, (**E**) a twisted paperfluidic device, and (**F**) a crumbled paperfluidic device. (**G**) SERS spectra of UA collected under 0 and 30% strains. (**H**) Infrared thermal image of a device placed on the hot plate at 45°C with a laser on. (**I**) SERS spectra of 100 μM UA collected at 21° and 45°C. Scale bars, 1 cm.

The soft, ultrathin, stretchable double-sided adhesive (180 μm thick) and PDMS (80 μm thick) encapsulated serpentine paperfluidics provide stretchability and flexibility of the wearable devices. All functional components described in Fig. 1C were assembled on a double-sided medical adhesive on a flexible, temporary paper liner (Fig. 6A). After removing the flexible paper liner, a freestanding device with a paperfluidic channel width of 2 mm was mounted on a mechanical stretcher to examine the stretchability (Fig. 6B). For reference, human skin has a linear response to tensile strain up to 15% and a failure stain at >30% (*59*). We did not observe any delamination between all functional layers in the device under 30% stretch (Fig. 6C). Under 60% stretch, a small tear appears at the edge of the paperfluidic layer (Fig. 6D). However, the plasmonic sensors are still in the same location due to the mechanical strain isolation provided by the serpentine paperfluidic layer. Twisted and crumbled paperfluidic devices further demonstrate the flexibility of the soft mechanical construction (Fig. 6, E and F). The change in the ratiometric SERS intensity of UA is 9% under 30% stretch compared to that without stretch (Fig. 6G). We also characterized the stability of SERS intensity at elevated temperatures. Figure 6H shows an infrared thermal image of a device placed on the hot plate at 45°C with a laser focused on the sensor. The temperature at the sensor location was 8°C higher than the surrounding temperature because of the plasmonic thermal effect resulting from a laser power of 35.5 mW. The change in the ratiometric SERS intensity of UA is 2% measured at 45°C compared to that at 21°C (Fig. 6I). These results demonstrate that the ratiometric SERS intensity is insensitive to mechanical deformation and temperature variations.

**Fig. 7.**
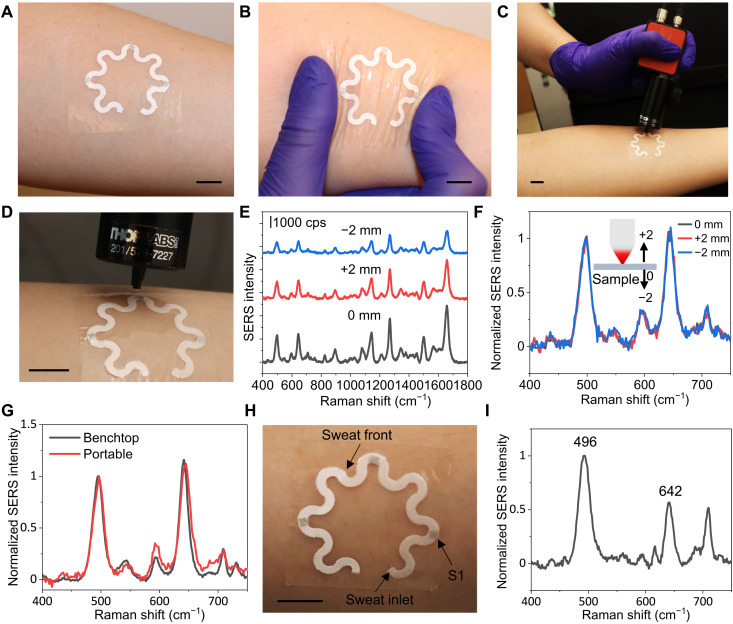
Application demonstration. Photographs of (**A**) a device conformally laminated on the forearm of a human subject, (**B**) under deformation, and (**C** and **D**) a portable Raman spectrometer with a flexible fiber probe for spectra collection. SERS spectra of 100 μM UA with the changing distance between the laser source and the plasmonic sensor (**E**) before and (**F**) after spectra normalization. (**G**) Comparison of SERS spectra collected with benchtop and portable spectrometers. (**H**) Photograph of the device after a healthy human subject wore it and exercised for 20 min. (**I**) SERS spectrum of the sweat collected from the sensor S1 in (H). Scale bars, 1 cm.

For proof of concept, we evaluated the performance of plasmonic paperfluidic devices for sweat collection and analysis in healthy human subjects. The device can be easily applied and comfortably worn at any body location due to its soft mechanical construction (Fig. 7A). The flexible and stretchable design can accommodate the skin deformation without device delamination and constraints in natural body motions (Fig. 7B). To enable the application of wearable plasmonic paperfluidic devices in point-of-care settings, we evaluated the accuracy of the UA quantification using a portable Raman spectrometer in comparison with a standard benchtop system. Figure 7C shows a photograph of the flexible fiber probe of a portable Raman spectrometer collecting SERS spectra from a plasmonic paperfluidic device laminated on the forearm of a healthy human subject. It is very robust to collect SERS signals using a handheld Raman spectrometer with an optimized cone-shaped probe when the human subject was instructed to keep the arm still for 10 s during each measurement (Fig. 7D). We optimized the probe length to achieve maximal SERS intensity when the probe contacts the sensor surface. The SERS intensity varies with the distance between the laser source and the plasmonic sensor (Fig. 7E). The SERS intensity reaches a maximum at the distance of 11 mm, and the intensity decreases by 40 and 60% when the laser probe moves up and down by 2 mm, respectively. However, the ratiometric SERS intensity remains the same with the offset laser focus (Fig. 7F). In addition, the probe opening diameter of 1 mm matches the sensor size to eliminate the misalignment (Fig. 7D). The misalignment between the laser and sensor could change the SERS intensity ratio (fig. S10). To eliminate laser-induced skin damage (*60*), a thin layer of carbon tape was sandwiched between plasmonic paper and double-sided adhesive to completely block a laser power of 65 mW (fig. S11). The temperature at the sensor location increases from 30.6° to 40.4°C within 5 s of laser exposure (29.4 mW), rises to the maximum temperature of 53°C, and then reaches equilibrium within 2 min (fig. S12). Because of the blocking effect of the carbon tape, the temperature increases only by 0.6°C on the skin-interfaced side after the laser exposure for 10 s. It takes 6 min to reach the maximum temperature of 34.8°C. In both cases, the temperatures dropped back to the original value within 1 min after the laser was turned off. These data suggest that each spectrum collection with laser power < 30 mW and exposure time ≤ 5 s keeps the temperature on the skin surface well below the safety limit set by the IEC 60601-1 standard (43°C). We further confirmed that the relative SERS intensity remained the same when the SERS spectra were collected with a benchtop and portable spectrometer (Fig. 7G). We collected the SERS spectrum of sweat after a healthy human subject ran for 20 min outdoors wearing a device on the forearm. The human subject did not experience any skin irritation or discomfort during the wear and after the device was detached. The sweat volume collected by the device was 13.7 μl (Fig. 7H). The quantified concentration of UA in sweat is 28 μM, which is consistent with the concentration of UA in the sweat of healthy individuals reported in the literature (*17*).

## DISCUSSION

The wearable plasmonic paperfluidic platform reported here enables sweat collection, transport, storage, and continuous, real-time, label-free biochemical analysis. Wearable sweat sensors relying on an enzymatic reaction or antibody-antigen interaction are limited by the poor chemical and environmental stability of the biorecognition elements. On the other hand, SERS provides the chemical fingerprint of sweat metabolites without involving any unstable biorecognition elements. In addition, the SERS signals are insensitive to the inevitable changes in mechanical strain on the wearable devices and operational temperature, thus eliminating the need for calibration associated with these changes during real-time, in situ analysis. This particular feature of the plasmonic sensor is advantageous compared to electrochemical sensors that require calibration for stable performance and wearable operations under various conditions.

For deducing actionable biochemical information from wearable sweat sensors, one needs to acquire and integrate data related to both sweat rate and analyte concentrations simultaneously. Simple paper microfluidic devices can accurately quantify sweat loss and sweat rate, and simultaneously, the plasmonic sensors quantify analyte concentrations. Wearable plastic and silicone microfluidic devices use the natural pressure created by sweat glands to drive sweat into hydrophobic channels (*10*, *17*, *61*). However, the gland secretory pressure could limit the capability of pushing resting sweat with low secretion rates into the devices and restrict quantifiable sweat volume due to hydraulic pressure loss (*61*). In contrast, chromatography paper can actively transport sweat through strong capillary force with well-defined flow characteristics without relying on the secretory pressure of the sweat glands. Harnessing the unique attributes of plasmonic paper, we established the engineering design guidelines of integrating various functional layers to form soft, flexible, and stretchable devices for comfortable wear at any body location.

In this work, the wearable plasmonic sensors enable the sensitive detection and quantification of UA in sweat at concentrations as low as 1 μM. Furthermore, we demonstrated the continuous monitoring of UA at varying concentrations that are physiologically and pathologically relevant in human sweat. Our design uses the ratiometric SERS intensity as a quantification approach to obviate the need for recalibration with different Raman spectrometers, thus facilitating the broad deployment of wearable plasmonic devices. At present, handheld Raman spectrometers are not well developed as the integrated electronic readout system for electrochemical sensors. Future advances in miniaturized and wearable optical readout systems will propel wearable plasmonic devices into the real world. Our future studies will optimize and evaluate the use of miniaturized Raman spectrometers for in situ sweat analysis. The device principles and sensing platform introduced here can be exploited to continuously measure other biomarkers in sweat or other biofluids such as saliva, and interstitial fluids, known to have small sample volumes, for disease diagnosis and health monitoring.

## MATERIALS AND METHODS

### Materials

All chemicals were used as received. Gold (III) chloride trihydrate (>99.9%, 520918), ascorbic acid (>99.0%, A5960), and CTAB (>99%, H5882) were obtained from Sigma-Aldrich. Sodium borohydride (98%, S678), silver nitrate (>99%, S181), double-sided carbon tape (5028581), and cellulose chromatography paper (Whatman no. 1 grade, 09-927-690) were obtained from Fisher Scientific. UA (A13346), l-tyrosine (99%, A11141), and l-phenylalanine (>99%, A13238) were obtained from Alfa Aesar. d-Glucose (4912-12) was purchased from MACRON Chemicals. PBS (10×, ultrapure grade) was obtained from Hoefer. Artificial sweat was obtained from Biochemazone. The purchased artificial sweat does not contain UA. Medical-grade double-sided adhesive tape (2477P) was obtained from 3M. PDMS elastomer (Sylgard 184) was purchased from Dow Corning. Type 1 deionized (DI) water (18.2 milliohm·cm) was used in all experiments and produced by Sartorius Arium Pro ultrapure water system.

### Synthesis of AuNRs

The AuNRs were prepared using a modified seed-mediated growth method (*48*, *49*). Briefly, the seed solution was first prepared by mixing 9.75 ml of 0.1 M CTAB and 0.25 ml of 10 mM HAuCl_4_ with 0.6 ml of an ice-cold 10 mM NaBH_4_ aqueous solution prepared within 30 min under 500 rpm stirring at 22°C. Separately, a growth solution was prepared by mixing 95 ml of 0.1 M CTAB with 5 ml of 10 mM HAuCl_4_ and then 1 ml of 10 mM silver nitrate solution and 0.55 ml of 0.1 M ascorbic acid. After gently mixing the growth solution, 0.12 ml of the seed solution was added to the growth solution to yield AuNRs. The AuNRs were aged for 12 hours to ensure full growth at room temperature. The AuNR solution was centrifuged at 8000 rpm for 10 min to remove excess chemical reagents for further usage.

### Preparation of AuNR paper and paper microfluidic device

A 2 cm–by–2 cm chromatography-grade filter paper was immersed in the AuNR solution with a longitudinal LSPR intensity of 2.5, 5, and 10 for 15 hours to allow AuNRs to uniformly adsorb on the paper surface. The longitudinal LSPR intensity was quantified from AuNR solution with an optical path length of 10 mm using an ultraviolet-visible (UV-vis) spectrophotometer (Shimadzu UV-1900). The as-synthesized AuNR solution was concentrated five times after centrifugation to obtain the AuNR solution with the longitudinal LSPR intensity of 5. Subsequently, AuNR paper was washed with DI water to remove loosely bound AuNRs and left naturally dry at room temperature (fig. S9). To remove CTAB from the AuNR surface, AuNR paper was immersed in 10 mM NaBH_4_ for 10 min, washed with DI water under running DI water for 15 s, and then dried in a desiccator for 2 hours at 22°C. Paperfluidic devices with different channel widths of 1 to 3 mm were prepared by cutting chromatography paper into desired serpentine shape using a paper cutting tool (Cricut maker, premium fine-point blade) with predesigned AutoCAD patterns. AuNR paper was cut into pieces of 2 mm–by–2 mm square or 1-mm-diameter circular shape. Last, double-sided carbon tape, paperfluidic layer, and AuNR paper were assembled on a medical-grade double-sided adhesive tape and then encapsulated with a thin PDMS film. The thickness of PDMS films was varied from 30 to 134 μm to investigate the effect of the PDMS thickness on SERS intensity. To fabricate PDMS films, parts A and B of Sylgard 184 silicone elastomer kit were mixed in a 10:1 weight ratio, degassed under vacuum for 15 min, and then spin-coated onto a polystyrene substrate. Spin coating the mixture at 1000 rpm for 5 min, 800 rpm for 1 min, and 500 rpm for 1 min yields the PDMS thickness of 30.0 ± 1.4 μm, 79.9 ± 3.7 μm, and 133.7 ± 1.0 μm, respectively (fig. S13). The variations in the thicknesses of the PDMS films fabricated in different batches are less than 5%.

### Characterization and measurements

The extinction spectra of AuNR aqueous solution were collected using a UV-vis spectrophotometer (Shimadzu UV-1900). Extinction spectra of AuNR paper were measured using a microspectrophotometer (CRAIC 308PV) connected with a Leica optical microscope (DM4M) with 20× objective in the range of 450 to 900 nm with 10 accumulations and an ∼0.2-s exposure time in the reflection mode. Transmission electron microscopy (TEM) images were collected with JEOL 1200 EX, and AuNR dimensions were estimated from TEM images using ImageJ. SEM images were recorded with an ultrahigh-resolution field-emission SEM (JEOL JSM-7500F). Raman spectra were collected with a benchtop Raman spectrometer (DXR Raman Microscope, Thermo Fisher Scientific) at the laser wavelength of 780 nm using a laser power of 20 mW and 10× objective. The spectra were measured in the wavelength range of 400 to 1800 cm^−1^ with an acquisition time of 60 s. SERS spectra were also collected with a portable Raman spectrometer (Wasatch Photonics Raman 785) with a laser wavelength of 785 nm and an acquisition time of 5 s. Infrared thermal images of freestanding devices or devices on a hot plate (SuperNuova+) were collected with a thermal imager (FLIR E54). Video recordings were collected to quantify the wicking rate of the paper microfluidic devices. ImageJ software was used to quantify the travel distances of the liquid, i.e., the central distance between the liquid front and the inlet of the serpentine paper, at different time points. Two replicates were performed in characterizing the device performance in both a continuous and time-sequenced mode of operation. The skin was washed with soap and running water and dried with a single-service paper before wearing a device. All experiments involving human subjects were conducted under approval from the Institutional Review Board at the Texas A&M University (project number 118141). One purpose of this study was to develop and demonstrate a wearable plasmonic paper–based microfluidic device for collecting and analyzing sweat. Human subjects were recruited from healthy adult volunteers. Briefly, healthy individuals were recruited, informed consent was obtained after the procedure, and the potential risks of the study were explained. The devices collected sweat from the forearm of human subjects, and then in situ analysis was performed using a portable Raman spectrometer.
