# Supplementary material for: Wearable plasmonic paper–based microfluidics for continuous sweat analysis
Source: Sci Adv. 2022 Mar 23;8(12):eabn1736. doi: 10.1126/sciadv.abn1736 (PMC8942375; doi:10.1126/sciadv.abn1736)
Supplement: Supplementary file 1 — Figs. S1 to S13 [file sciadv.abn1736_sm.pdf]

Science Advances
AAAS

Supplementary Materials for

Wearable plasmonic paper–based microfluidics for continuous sweat analysis

Umesha Mogera, Heng Guo, Myeong Namkoong, Md Saifur Rahman,
Tan Nguyen, Limei Tian*

*Corresponding author. Email: ltian@tamu.edu

Published 23 March 2022, *Sci. Adv.* **8**, eabn1736 (2022)
DOI: 10.1126/sciadv.abn1736

This PDF file includes:

Figs. S1 to S13

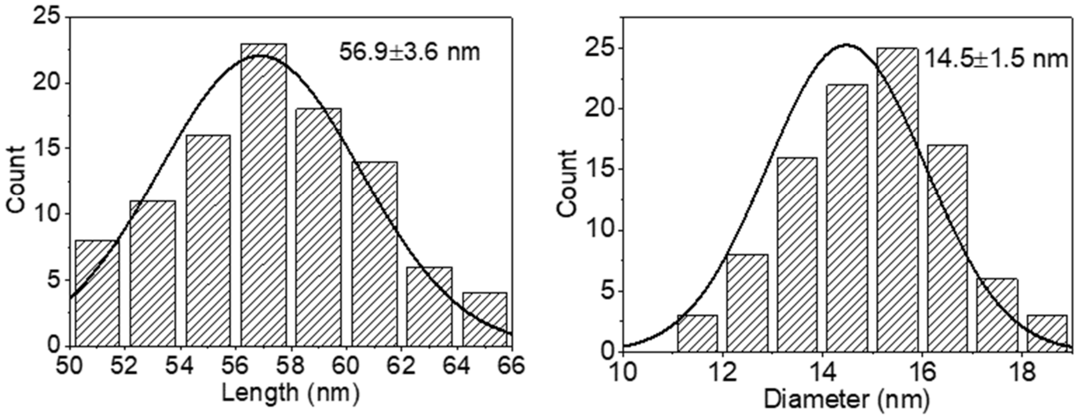

Figure S1. Size distribution of the as-synthesized AuNRs.

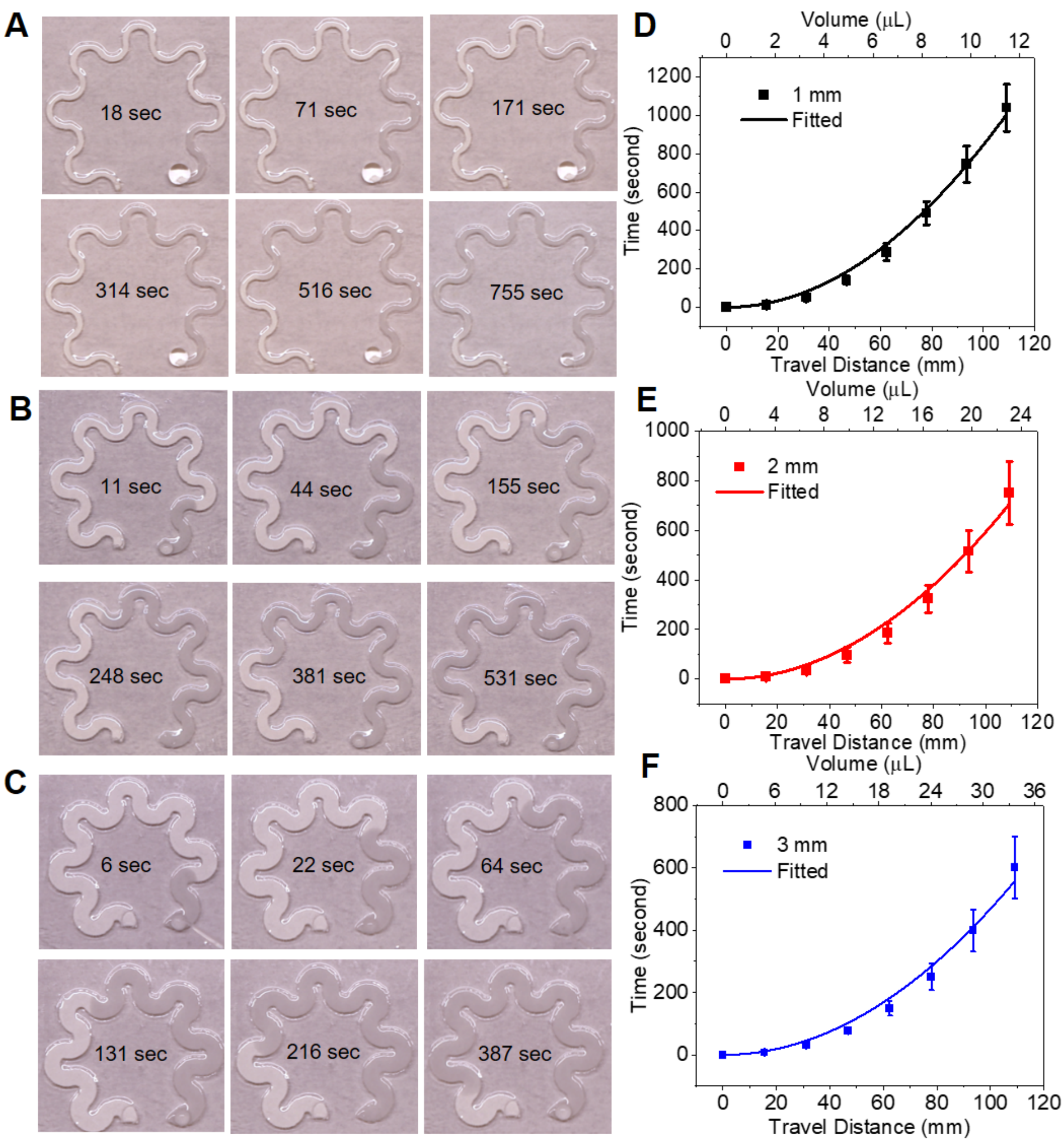

Figure S2. **Characterization of paper microfluidic devices for sweat loss/rate quantification.** Photographs of the paperfluidic devices with a varying width of (A) 1 mm, (B) 2 mm, and (C) 3mm collected at different time points of continuous flow after introducing a drop of water to the inlet. Correlations between liquid travel distance, corresponding liquid uptake volume of the microfluidic paper with the channel width of (D) 1 mm, (E) 2 mm, and (F) 3 mm, and time.

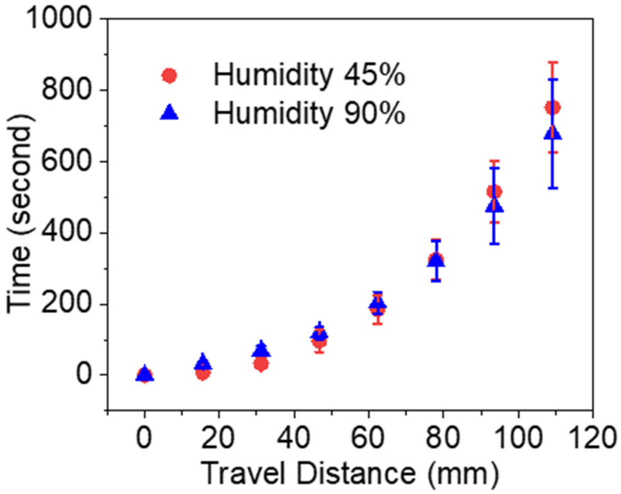

Figure S3. **Liquid travel distance comparison of the microfluidic paper under different humidity.** The microfluidic paper channel width is 2 mm, and the relative humidities are 45% and 90%.

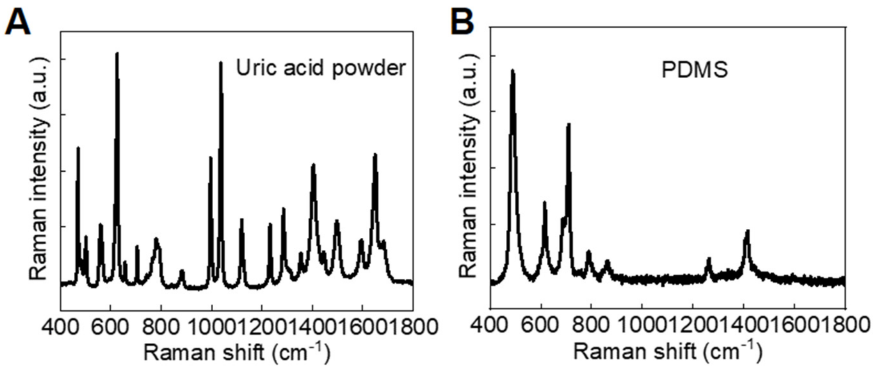

Figure S4. Raman spectra of UA powder and a PDMS film.

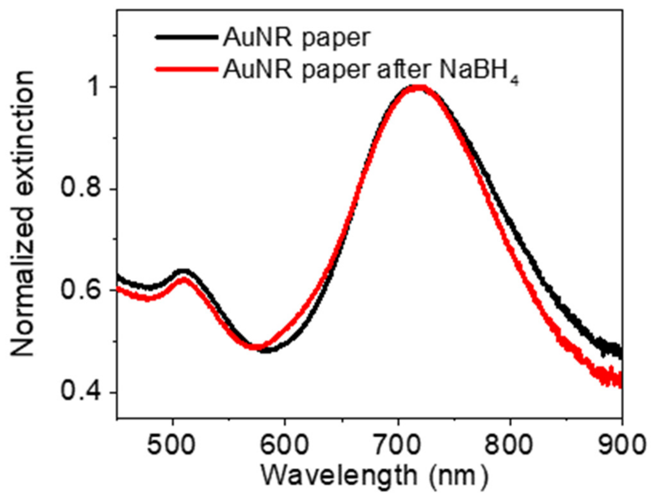

Figure S5. The normalized extinction spectra of AuNR paper before and after $NaBH_4$ treatment.

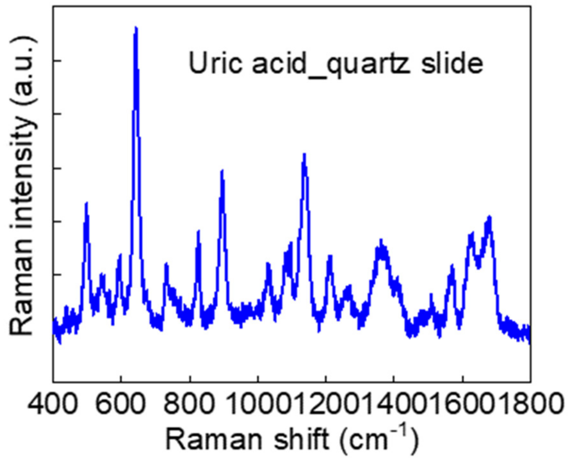

Figure S6. SERS spectrum of UA collected on AuNR paper covered with a quartz slide.

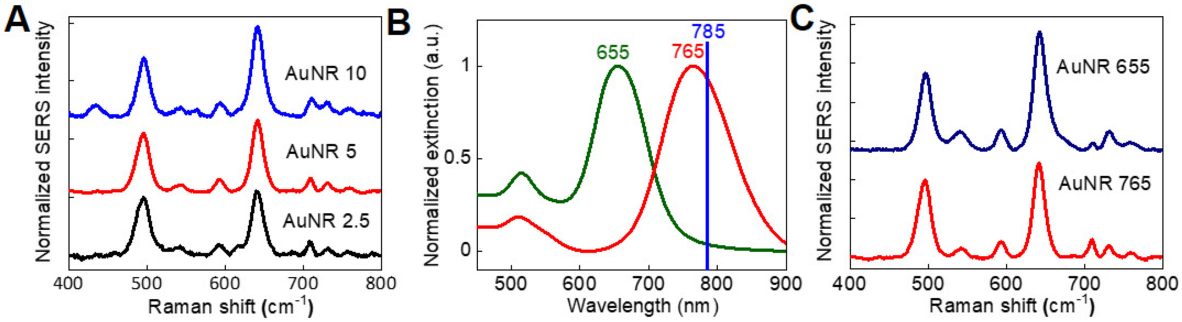

Figure S7. SERS intensity comparison of UA collected from AuNR papers with different AuNR sizes and densities. (A) SERS spectra of 100 μM UA collected from the AuNR paper prepared using AuNR solution with the longitudinal LSPR wavelength of 765 nm and the extinction intensity of 2.5, 5, and 10. (B) Extinction spectra of AuNR solution with the longitudinal LSPR wavelength of 655 nm and 765 nm. The laser excitation wavelength is 785 nm. (C) SERS spectra of UA collected from the AuNR paper prepared using the AuNR solution with the longitudinal LSPR wavelength of 655 nm.

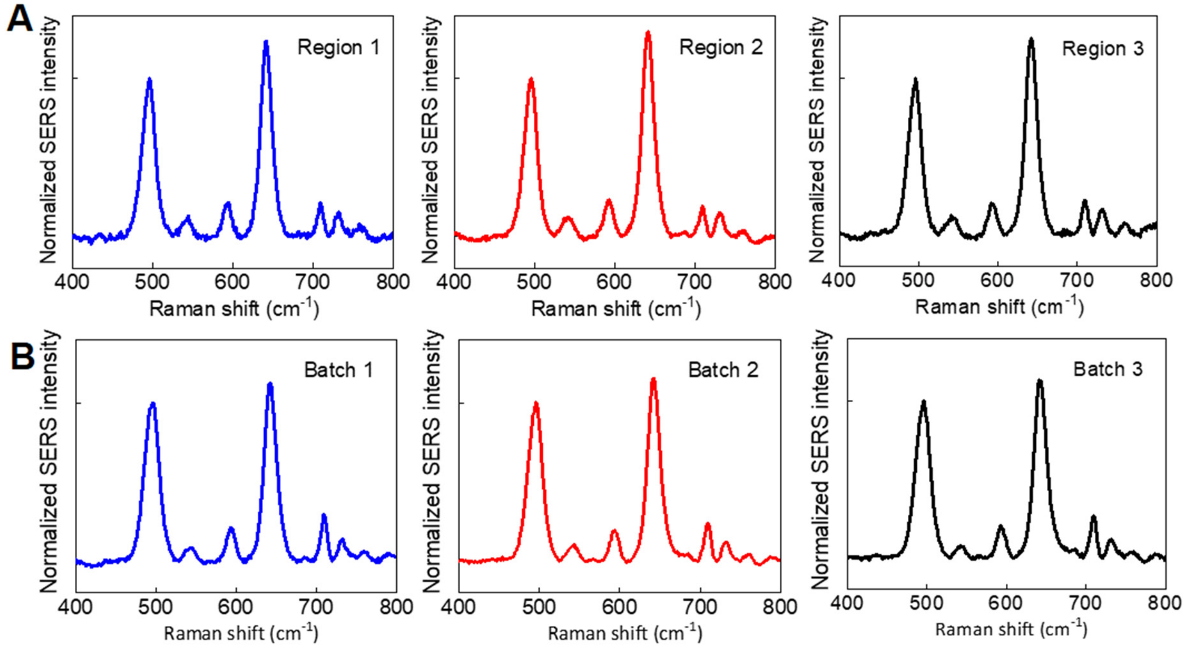

Figure S8. SERS spectra uniformity characterization. SERS spectra of 100 μM UA collected from (A) different regions of the same AuNR paper and (B) different batches of AuNR paper.

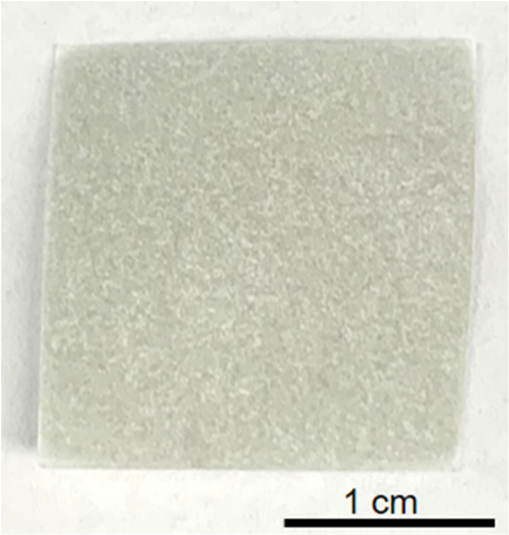

Figure S9. Photograph of 2 cm x 2 cm AuNR paper.

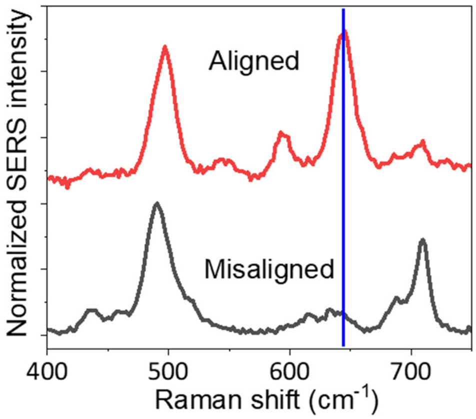

Figure S10. SERS spectra of UA collected with the laser aligned and misaligned with the sensor.

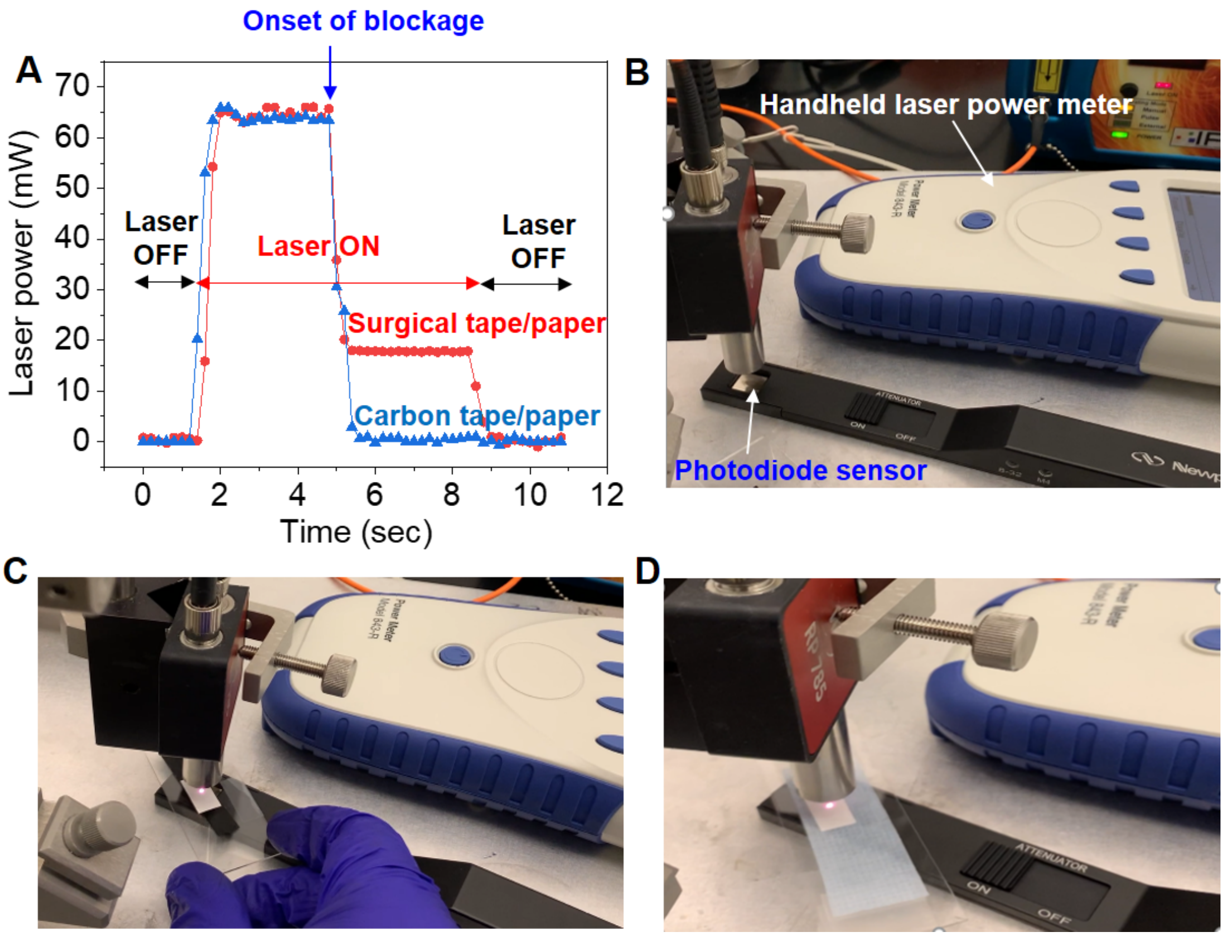

Figure S11. Characterization of a black carbon adhesive as a laser blocker. (A) The measured laser power of the portable Raman spectrometer before and after blocking with a chromatography paper laminated on two types of adhesive, including black carbon double-sided adhesive and 3M Micropore surgical tape. A carbon tape/paper completely blocks the laser power of 65 mW, and a surgical tape/paper blocks 72% of the laser power. (B) Photography of the laser probe pointing to the photodiode sensor with the laser off. Photographs of (C) a chromatography paper laminated on black carbon tape and (D) chromatography paper laminated on a flexible surgical tape mounted on a quartz slide placed between the laser and the photodiode sensor.

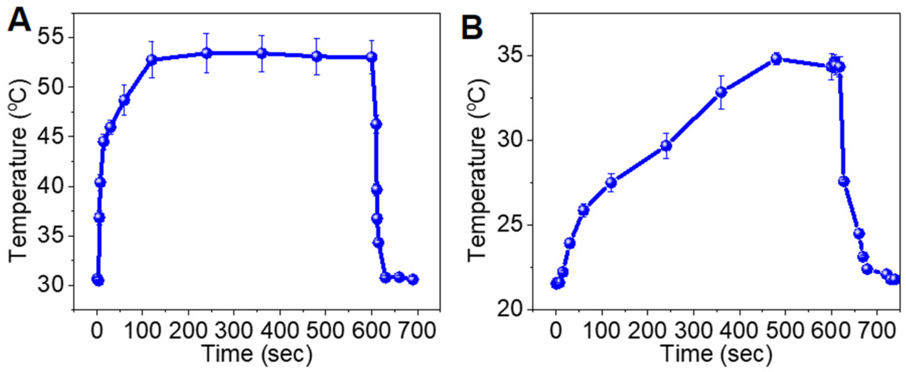

Figure S12. Temperature changes at sensor locations as a function of time. The data were collected using a thermal camera (A) on the sensor surface with the device kept on a hotplate at 31°C (simulating skin temperature) and (B) on the skin-interfaced side below the sensor with the laser power of 29.4 mW.

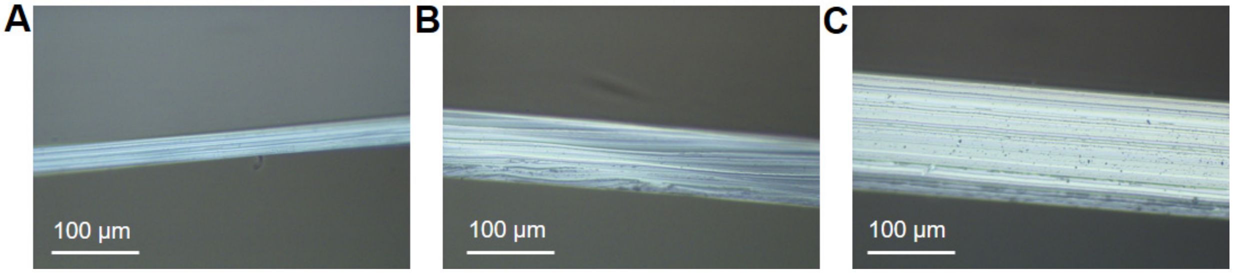

Figure S13. Optical microscope images of the cross-sections of the PDMS films. The films were prepared by spin coating the Sylgard 184 mixture with 10:1 ratio (A) at 1000 rpm for 5 minutes, (B) at 800 rpm for 1 minute, and (C) at 500 rpm for 1 minute.
